# Which Multimodal Physiotherapy Treatment Is the Most Effective in People with Shoulder Pain? A Systematic Review and Meta-Analyses

**DOI:** 10.3390/healthcare12121234

**Published:** 2024-06-20

**Authors:** Maria Aguilar García, Ana González Muñoz, José Javier Pérez Montilla, Daniel Aguilar Nuñez, Dina Hamed Hamed, Leo Pruimboom, Santiago Navarro Ledesma

**Affiliations:** 1 Biomedicine PhD Program, Faculty of Health Sciences, University of Granada, 18071 Granada, Spain; mariaaguilar92@correo.ugr.es; 2 Clinical Medicine and Public Health PhD Program, Faculty of Health Sciences, University of Granada, 18071 Granada, Spain; agonzalezm@correo.ugr.es (A.G.M.); perezmontilla@correo.ugr.es (J.J.P.M.); dhamed@correo.ugr.es (D.H.H.); 3 Department of Nursing and Podiatry, Faculty of Health Sciences, University of Malaga, 29071 Malaga, Spain; daguilarn.tic@gmail.com; 4 University Chair in Clinical Psychoneuroimmunology, University of Granada and PNI Europe, 52004 Melilla, Spain; leo@cpnieurope.com; 5 Department of Physical Therapy, Faculty of Health Sciences of Melilla, University of Granada, 52004 Melilla, Spain

**Keywords:** shoulder pain, chronic pain, physical therapy, physiotherapy, multimodal treatment, exercise, manual therapy

## Abstract

The study aimed to determine if combined physiotherapy treatments offer additional benefits over exercise-only programs for shoulder pain and to identify the most effective combined treatment. A systematic review, registered in PROSPERO (CRD42023417709), and meta-analyses were conducted. Quality analysis was performed using the PEDro scale on randomized clinical trials published from 2018 to 2023. Twenty articles met the inclusion criteria. The most commonly used combination was exercise plus manual therapy, without being statistically superior to exercise alone. The meta-analysis indicated that combining exercise with low-level laser therapy (mean difference of −1.06, 95% CI: −1.51 to −0.60) and high-intensity laser therapy (mean difference of −0.53, 95% CI: −1.12 to 0.06) resulted in the greatest reduction in SPADI scores. Adding manual therapy provided limited additional benefit (mean difference of −0.24, 95% CI: −0.74 to 0.27). Progressive exercise with advice or telerehabilitation yielded modest improvements. The multimodal meta-analysis for DASH scores showed significant improvement (mean difference of −1.06, 95% CI: −1.51 to −0.60). In conclusion, therapeutic exercise is the cornerstone of shoulder pain treatment, with the addition of laser therapy showing substantial benefits. Manual therapy and educational interventions offer some benefits but are not consistently superior. More rigorous studies are needed.

## 1. Introduction

Shoulder pain is a pathology that is frequently found in clinical practice. It has an approximate incidence of 10 per 1000 cases in primary care [1,2], a prevalence of 12% in physiotherapy services [3], and a lifetime prevalence of up to 66.7% [4]. Therefore, it would be reasonable to conclude that shoulder pain is a significant illness in the general population and that it probably represents the most common musculoskeletal problem after back and neck pain [5].

There are multiple causes that can generate pain in the shoulder region, with the most common being rotator cuff tendinopathy/shoulder impingement syndrome [6] or subacromial entrapment syndrome or subacromial pain, which it is also known by [7]. Although many authors use different diagnostic labels, current evidence suggests that it is more appropriate to direct research lines to talk about the pathology as general shoulder pain in order to avoid diagnostic errors [8]. This pathology is seen more frequently in women and increases with age [9]. In the long run, pain causes a limitation of movement, functional deficit, and, therefore, a restriction in daily living activities [10,11]. 

The treatment of this pathology is mostly conducted through conservative interventions [12] that aim to reduce joint pain and stiffness, improve muscle strength, prevent the progression of problems, optimize shoulder function, and enable the person suffering from this condition to resume their daily activities as soon as possible [11,13,14,15]. There are numerous different types of treatment that have been undertaken, among which are exercise programs for the rotator cuff and the scapular region, manual therapy techniques, modification of daily activities, and a wide variety of other physiotherapy methods such as electrotherapy, ultrasound, and laser [14,16].

Therapeutic exercise, which has been shown to improve clinical symptoms in most patients, is the most-used treatment. Although many studies are conducting research into which type of exercise or combination of exercise may be the most effective [17,18,19,20,21,22,23,24], and there are recent systematic reviews on which conservative approach is the most effective [15,25], there are no studies looking into whether therapy combined with other techniques may be more effective than exercise alone, and/or which combination of treatments may be the most effective.

Therefore, the aim of this systematic review will be to review all articles that address the treatment of shoulder pain using different physiotherapy techniques in order to establish which treatment, in combination with exercise, may be the most effective. Additionally, the quality of the included studies will be assessed.

## 2. Methods

### 2.1. Data Sources and Searches

This systematic review was performed in accordance with PRISMA guidelines [24] and is registered in the PROSPERO register of systematic reviews (CRD42023417709). The searches were conducted during January, February, March, April, and May 2023. The online databases selected were PubMed, Web of Sciences (WoS), and Scopus. The searches were conducted by two authors, as well as the risk assessment and selection of articles. To avoid disagreements, all search criteria, evaluation, and article selection were agreed upon before starting the study.

In accordance with the Population, Intervention, Comparison, Outcome, and Study Design (PICOS) strategy, the search aimed to find randomized clinical trials (RCTs) (S) by comparing the use of different combined physiotherapy treatments (C) in patients with shoulder pain (P) in order to establish which is the most effective individual or multimodal treatment (I).

#### 2.1.1. PubMed

The search strategy was: “Shoulder” AND “Pain” AND (“Exercise” OR “Physical Therapy” OR “Manual Therapy” OR “Multimodal Treatment” OR “Education” OR “Pain Education” OR “Neuroscience Education”).

Filters used were:

- Full text.
- Type of article: Clinical Trial and Randomized Controlled Trial.
- Language: English and Spanish.
- Population: adults, 19+ years.
- Date of publication: from 2018–2023.

#### 2.1.2. Web of Sciences (WoS)

The search strategy was: TI = (“Shoulder” AND “Pain” AND (“Exercise” OR “Physical Therapy” OR “Manual Therapy” OR “Multimodal Treatment” OR “Education” OR “Pain Education” OR “Neuroscience Education”)).

Filters used were:

- Full text.
- Type of article: Clinical Trial and Randomized Controlled Trial.
- Language: English.
- Population: adults.
- Date of publication: from 2018 to 2023.

#### 2.1.3. Scopus

The search strategy was: “Shoulder” AND “Pain” AND (“Exercise” OR “Physical Therapy” OR “Manual Therapy” OR “Multimodal Treatment” OR “Education” OR “Pain Education” OR “Neuroscience Education”).

Filters used were:

- Full text.
- Type of article: Clinical Trial and Randomized Controlled Trial.
- Language: English and Spanish.
- Population: adults.
- Date of publication: from 2018–2023.

### 2.2. Study Selection

#### 2.2.1. Inclusion Criteria

The following inclusion criteria had to appear in the searches:

- Studies published from January 2018–May 2023.
- English or Spanish language publication.
- Type of article: RCTs.
- Studies comparing the effectiveness of different combined physiotherapy treatments.
- Population: human adults (>18 years).
- Individuals with chronic shoulder pain (duration > 3 months).
- Full text available.

#### 2.2.2. Exclusion Criteria

- Studies repeated in the databases.
- Studies conducted in humans with neurological disorders, acute shoulder pathologies (fractures, full-thickness rotator cuff tear, etc.), shoulder surgery and cancer.
- Studies that include only treatments such as steroid injection, corticosteroid injection, or any analgesic pill.

### 2.3. Article Selection

Once the search was conducted, full-text articles that had a title in accordance with the objective of this systematic review were selected. Next, each full-text article was analyzed to verify if it met the inclusion criteria. Finally, the methodological quality of the studies was checked.

### 2.4. Evaluation of the Methodological Quality

In order to evaluate the methodological quality of the articles selected, the PEDro scale, translated and adapted to Spanish, was used [26]. This scale consists of 11 items that examine the internal and external validity of the study. The scale has ranges from 0 to 10, where each item scores one point, except for the first one that is not included [26]. Studies that obtain between 9 and 10 points are of excellent methodological quality; those with six-to-eight points have good methodological quality; those with four-to-five points have fair quality; and, finally, those that have less than four points have poor methodological quality [26].

### 2.5. Statistical Analyses

The meta-analysis employed the standardized mean difference as the outcome measure and utilized a random-effects model for data fitting. The extent of heterogeneity tau^2^) was determined using the restricted maximum-likelihood estimator. Alongside the τ^2^ estimate, the Q-test for heterogeneity and the I^2^ statistic were also reported. When any degree of heterogeneity is identified (τ^2^ > 0, irrespective of the Q-test results), a prediction interval for the true outcomes is provided. Studentized residuals and Cook’s distances are utilized to assess whether studies are outliers or influential within the model. Studies with a studentized residual exceeding the 100 × (1 − 0.05/(2 × k))the percentile of a standard normal distribution are deemed potential outliers (applying a Bonferroni correction with two-sided alpha = 0.05 for k studies included in the meta-analysis). Studies with a Cook’s distance greater than the median plus six times the interquartile range of the Cook’s distances are considered influential. Funnel plot asymmetry is examined using the rank correlation test and the regression test, with the standard error of the observed outcomes serving as the predictor.

## 3. Results

### 3.1. Search Results

**Figure 1:**
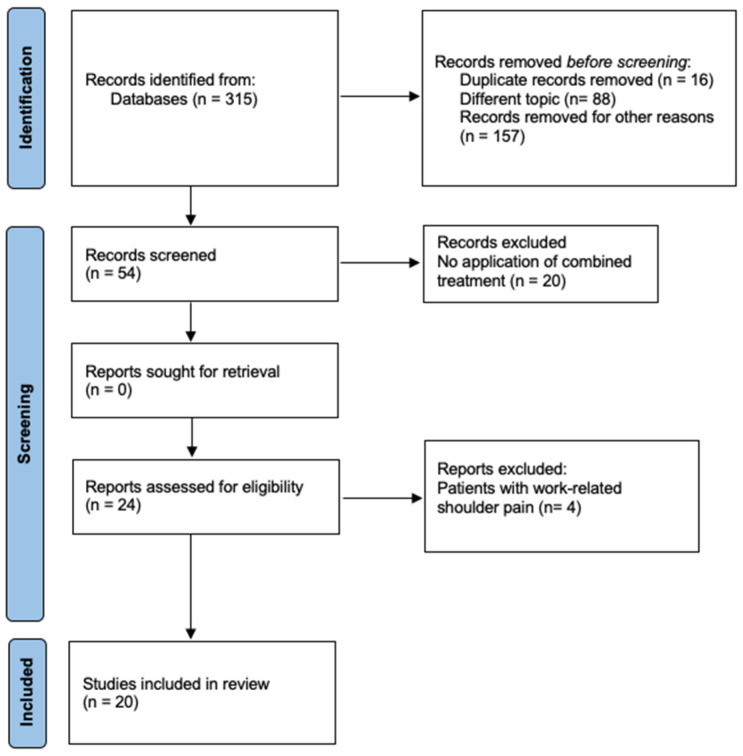
Flowchart of article selection following the PRISMA 2020 statement [27].

A total of 5745 hits were found after the initial search. Once the filters were applied, 315 articles were obtained; after a new filter, 42 articles remained to be read and analyzed to check if they met the inclusion and exclusion criteria. Twenty articles were eliminated for applying non-combined physiotherapy treatments, and the other four articles were also rejected because they treated patients with work-related shoulder pain. Finally, 20 articles that met the inclusion criteria were included in this systematic review. This selective process is shown in Figure 1 by means of a flowchart.

### 3.2. Methodological Quality Evaluation

Once the articles were selected, the quality analysis was conducted using the PEDro scale. The results are shown in Table 1.

### 3.3. Study Characteristics

A total of 20 articles were reviewed with the number of patients in each study, varying from 24 [37] to 708 [32], which added up to a total of 2385 patients. All of the patients who were suffering from shoulder pain had different diagnostic labels.

Most studies referred to the shoulder pain as “subacromial impingement syndrome” (SIS) [28,39,40,41,42,44,47], “subacromial shoulder impingement” (SSI) [29], or “subacromial pain syndrome” (SAPS) [30,45,48]. Some studies used generic labels such as “shoulder pain syndrome” (SPS) [33,35,37], “shoulder pain”, [38] or “no specific shoulder pain” [46]. Three studies referred to diagnostic labels that recently appeared, namely “rotator cuff disorder/syndrome” [32,34] and, more frequently, “rotator cuff-related shoulder pain” [34]. Finally, only three studies included patients who were diagnosed with “chronic shoulder pain” [31,43,49], although the patients in the rest of the studies also suffered from long-term shoulder pain.

The mean age varied between 30.93 ± 10.87 (Naranjo-Cinto et al., 2022) [46] and 61.3 ± 8.9 [42].

Different physiotherapy techniques were used in isolation or in combination to treat the groups in the 20 reviewed articles; the mean duration of treatment was between 8 and 12 weeks.

The exercise was performed in all the studies, except the one by Gomes C et al., 2018 [40], which was used for at least one of the groups in each study, alone or in combination with other physiotherapy techniques such as conventional physiotherapy techniques (ultrasound, hot application, TENS, etc.) [28,34,47,50,51], passive mobilizations and different manual therapy techniques [29,33,34,40,46,50], electrical dry needling and IFC [30], photobiomodulation therapy and suprascapular nerve radiofrequency [31], and corticoid injections [35] and different types of laser therapies [41,42].

The most-used outcome measures were those intended to assess pain, functionality, disability, and shoulder range of motion (ROM), which include the visual analog scale (VAS), shoulder pain and function disability index (SPADI), disability of the arm, shoulder and hand (DASH), numeric pain rating scale (NPRS), multimodal rating of change scale (GROC), Constant–Murley score (CMS), Pain Catastrophizing Scale (PCS), and Pain Self-Efficacy Questionnaire (PSEQ).

The main characteristics of each study, including patient characteristics, sample size, treatments performed, measurements taken, and results obtained, are shown in Table 2.

### 3.4. Data Synthesis

The different ways in which the varied therapies have been studied by each of the reviewed authors are briefly presented below.

Tahran O et al., 2020 [28] studied whether “modified cross-body stretch” (MCS) and modified sleep stretch (MSS) exercises (five repetitions each for 30 s five times a week for four weeks) were more beneficial when added to conventional physiotherapy treatment, which consisted of electrotherapy plus strengthening exercises for the musculature of the scapular, rotator cuff, and deltoid regions, plus stretching of the trapezius muscle (five sessions per week for 4 weeks). Their study showed that the inclusion of MCS and MSS stretches led to a greater improvement in outcomes (*p* < 0.005), although there were no significant differences between one type of stretch and the other.

Land H et al., 2019 [29] showed that manual therapy techniques, consisting of mobilizing the costovertebral joints on the symptomatic side (T1–T7 levels) for 6 weeks (G1) and mobilizing the glenohumeral joint with rotator cuff musculature massage (G2) when combined with home-based exercise (thoracic spine extensions for G1 and passive cross adduction stretch for G2) two times a day for 12 weeks, were as effective as each other and superior to the application of US alone.

Dunning J et al., 2021 [30] compared the efficacy of two combined physiotherapy treatments. In the first group, manipulative techniques at the lower cervical level (C4–C6), cervicothoracic level (C7–T3), mid-thoracic level (T4–T9), and the costovertebral joints (1–3) were combined with the application of electropuncture. In the second group, passive mobilization techniques (glenohumeral, acromioclavicular, and scapular joints) were combined with muscle-strengthening exercises, stretching exercises, and the application of IFC in the area of the subacromial space. The results revealed that both groups improved but that there was a statistically significant difference in favour of the group that received spinal manipulation and electropuncture techniques.

Ökmen B et al., 2017 [31] compared the effect of photobiomodulation plus exercise treatment with radiofrequency plus exercise treatment. The exercise protocol consisted of Codman exercises, stretching and strengthening exercises, and exercises to gain range of motion, performed once a day for 2 weeks. Both groups improved their pain and function values (*p* < 0.05), but there were no statistically significant differences between them.

Hopewell S et al., 2021 [32] studied whether progressive exercise was superior to best practice advice. Four groups were formed where the first received progressive exercise alone, the second received tips for best practices, the third had an injection of corticosteroids administered plus progressive exercise, and the fourth had an injection of corticosteroids plus more tips for best practices. The results showed that exercise was not superior to advice. Although all groups improved, at 8 weeks, it was observed that the injection of corticosteroids produced a greater reduction in pain in the groups that received it.

Eliason A et al., 2021 [33] sought to test whether adding manual therapy to guided exercise in patients with subacromial pain syndrome was more beneficial than performing it alone. The guided exercise consisted of 12 weeks of scapular retraction, adduction, and outward rotation exercises together with stretching of the upper trapezius and pectoral muscle. Group 1 received joint mobilizations plus guided exercise for 6 weeks, Group 2 received only guided exercise, and the third group was the control group. All groups improved significantly, and it appears that adding manual therapy is more effective in relieving symptomatology in the short term.

Menek B et al., 2019 [34] studied the difference in the effect of applying pain-free Mulligan mobilization techniques to exercise, TENS, and US versus applying traditional physiotherapy techniques to exercise, TENS, and US in patients with rotator cuff syndrome. Both groups performed the same exercises, which consisted of Codman exercises, internal and external rotation exercises, flexions, and extensions aimed at improving the elasticity of the shoulder capsule for 6 weeks (five times per week). The author concluded that, although both groups improved, the group receiving Mulligan mobilization techniques obtained more significant improvements compared to the other group (*p* < 0.05).

Roddy E et al., 2021 [35] examined the effectiveness of individualized, progressive, therapist-guided exercise in addition to ultrasound-guided and non-ultrasound-guided corticosteroid injections versus non-individualized, non-progressive exercise (following an informative pamphlet) along with ultrasound-guided and non-ultrasound-guided corticosteroid injections. The results showed that both groups improved with no significant differences between ultrasound-guided and non-ultrasound-guided infiltration. However, the groups that received guided and individualized exercise obtained significant differences compared to those that followed the exercises shown in the pamphlet (*p* < 0.05).

Malliaras P et al., 2020 [36] used patients suffering from related rotator cuff shoulder pain to compare the effectiveness of only providing advice on the pathology of the condition and the habits to be modified, versus offering advice and care recommendations (including pain education) together with 12 weeks of progressive exercise with adapted loads. Additionally, there was another group that combined advice with care (exercises and pain education) and telerehabilitation sessions in which educational tasks were carried out and self-care exercises were taught. It was observed that all groups improved but that the improvements were greater in the groups that, in addition to advice, received recommended care and telerehabilitation.

The article by De Oliveira A et al., 2022 [37] sought to verify whether the UT, MT, LT, and SA muscles benefited from the inclusion of EMG-biofeedback to exercise performed with adapted loads for 8 weeks, in patients with subacromial pain syndrome. The study concluded that both groups improved without significant differences, but at the Week 8 measurement, the group that had EMG-biofeedback obtained a significant improvement in the NPRS variable compared to the other group (*p* = 0.01).

Santello G et al., 2020 [38] compares performing an exercise program executed 3 days a week for 2 months versus treatment based on advice and self-care. The exercise program included stretching of the pectoral, UT, and posterior and lower structures of the shoulder, joint mobilizations, and strengthening of the rotator and SA muscles. The group that performed the exercise program obtained significant improvements in all variables when compared to the group that received self-care advice (*p* < 0.05).

Moslehi M et al., 2021 [39] analyzed the effectiveness of including feedback in the treatment of patients with shoulder impingement syndrome. In this study, three groups were established. Group 1’s treatment consisted of exercises that focused on the scapular region (stretching, strengthening of the muscles, and flexibility of the joint) while, at the same time, receiving tactile and verbal feedback from the physiotherapist in order to properly execute the exercises. Group 2 only performed the exercises focused on the scapular region, and Group 3 was the control group. The study concluded that the group that received verbal and tactile feedback showed significant differences (*p* < 0.05) in their results with greater improvements in all variables.

Gomes C et al., 2018 [40] compared the use of diadynamic currents in combination with manual therapy techniques versus the use of these techniques independently in patients with shoulder impingement syndrome, with a treatment duration of 8 weeks in each case. Although all groups improved (*p* < 0.05), the group that received the combined treatment showed more significant improvements than the other groups (*p* < 0.05).

Alfredo P et al., 2021 [41] studied the efficacy of low-level laser therapy in patients with SIS by comparing three groups. The first group received low-level laser therapy plus exercise, the second exercise alone, and the third laser alone. The three groups improved, and the group receiving the combination of low-level laser treatments plus exercise showed significant differences from the rest of the groups (*p* < 0.05).

Aceituno–Gómez J et al., 2019 [42] studied the efficacy of high-intensity laser therapy in patients with SIS. The patients were divided into two groups. The first group received high-intensity laser therapy plus exercise, while the second received placebo laser therapy plus exercise. Both groups improved significantly (*p* < 0.05), and there were no significant differences between them (*p* > 0.05).

Ingwersen K et al., 2019 [43] showed no differences between groups when comparing the effectiveness of treatment with psychomotor therapy plus exercise over exercise alone in patients with chronic shoulder pain (*p* > 0.05).

Gutiérrez–Espinoza H et al., 2019 [44] concluded that adding pectoralis minor stretches to exercise programs in patients with SIS has no extra benefit in terms of improving pain and function, although it does improve the pectoralis minor index.

In the study by Ribeiro D et al., 2022 [45], the effectiveness of a standardized exercise treatment was compared with the performance of personalized treatments that included both manual therapy techniques and specific exercise to re-establish mobility patterns, normalize weak muscles, etc. in 28 patients with PFS. Both groups underwent 8 weeks of treatment, with both obtaining improvements with no significant differences between their results.

Naranjo–Cinto F et al., 2022 [46] studied the use of manual therapy techniques, which used rhythmic mobilizations in the glenohumeral joint and in the thoracic spine (at the level of the third rib on the symptomatic side) plus exercise versus the use of exercise plus placebo mobility techniques. Both treatments lasted 5 weeks. All groups improved with no significant differences between them.

Finally, Alanazi A et al., 2022 [47] studied if better results were obtained by the addition of upper limb grip strengthening exercises to stretching exercises (posterior shoulder muscles, pectoral, etc.) and US. The article showed that both groups improved, but the results of the group that included gripping exercises were statistically significant.

### 3.5. Meta-Analysis

To determine which multimodal physiotherapy treatment is most effective for managing shoulder pain, a meta-analysis was conducted. To ensure sufficient methodological quality, meta-analyses were performed using the values of the two most commonly used variables to quantify the effectiveness of the treatments, which were SPADI and DASH. Additionally, using each of these variables, the different studies were analyzed and grouped according to the type of intervention applied. Thus, for the SPADI variable, a general meta-analysis and other subgroup meta-analyses were conducted for studies that implemented exercise, laser therapy, and manual therapy. For the DASH variable, the meta-analysis was performed only for studies that implemented exercise interventions, as only one study applied laser therapy [42], and only the study by Menek B et al., 2019 [34] implemented manual therapy.

#### 3.5.1. Multimodal Meta-Analysisg

**Figure 2:**
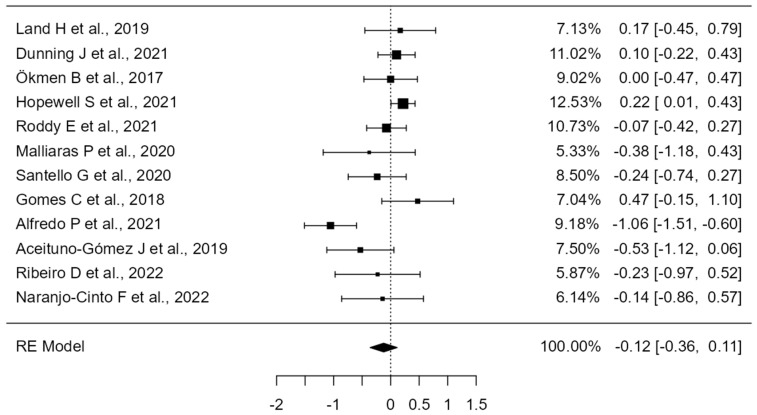
Forest Plot. Multimodal Meta-Analysis for shoulder pain and disability (SPADI). The small boxes with the squares represent the point estimate of the effect size and sample size. The lines on either side of the box represent a 95% confidence interval (CI) [29,30,31,32,35,36,38,40,41,42,45,46].

**Figure 3:**
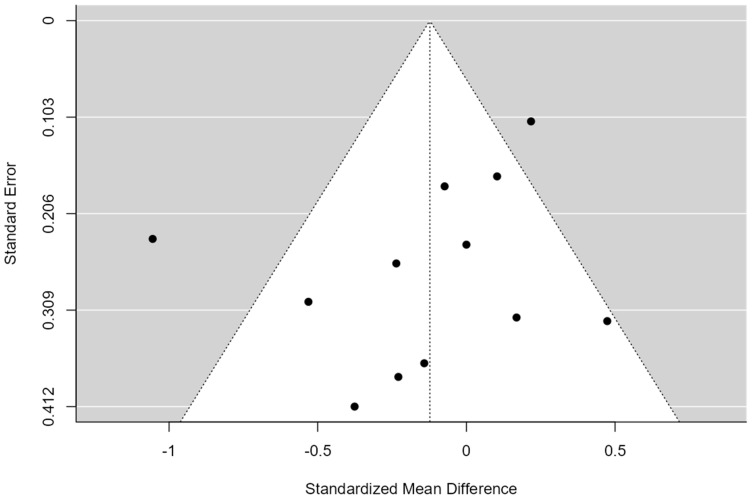
Funnel Plot of Multimodal Meta-Analysis for shoulder pain and disability (SPADI)**.** The plot is centered on a fixed effect summary estimate, the outer dashed lines indicate the 95% confidence interval of the fixed effect estimates. Symmetry is apparent when all studies are randomly dispersed around the dashed vertical line. Dark circles represent individual studies included in the meta-analyses.

A total of k = 12 studies were included in the analysis. The observed standardized mean differences ranged from −1.05 to 0.47, with the majority of estimates being negative (58%). The estimated average standardized mean difference based on the random-effects model was −0.12 (95% CI: −0.36 to 0.11). Therefore, the average outcome did not differ significantly from zero (z = −1.01, *p* = 0.31). According to the Q-test, the true outcomes appear to be heterogeneous (Q(11) = 32.73, *p* = 0.00, tau^2^ = 0.10, I^2^ = 67.16%). A 95% prediction interval for the true outcomes is provided by −0.80 to 0.55. Hence, although the average outcome is estimated to be negative, in some studies, the true outcome may in fact be positive. An examination of the studentized residuals revealed that one study had a value larger than ±2.86 and may be a potential outlier in the context of this model. According to the Cook’s distances, one study could be considered to be overly influential (see Figure 2). Neither the rank correlation nor the regression test indicated any funnel plot asymmetry (*p* = 0.24 and *p* = 0.49, respectively) (see Figure 3).

#### 3.5.2. Manual Therapy Treatment for Shoulder Pain and Disability (SPADI)

**Figure 4:**
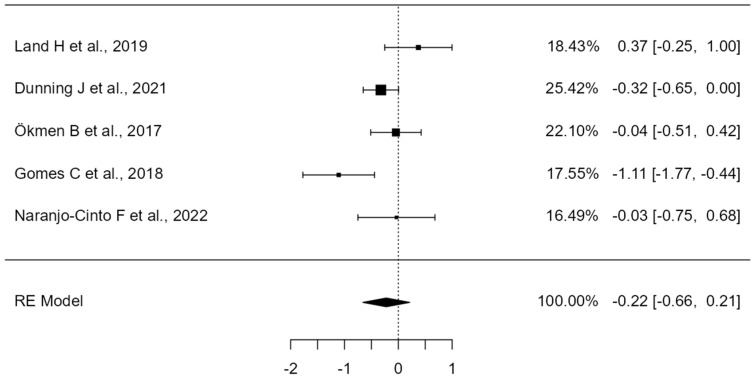
Forest Plot of manual therapy treatment for shoulder pain and disability (SPADI). The small boxes with squares represent the point estimate of the effect size and sample size. The lines on either side of the box represent a 95% confidence interval (CI) [29,30,31,40,46].

**Figure 5:**
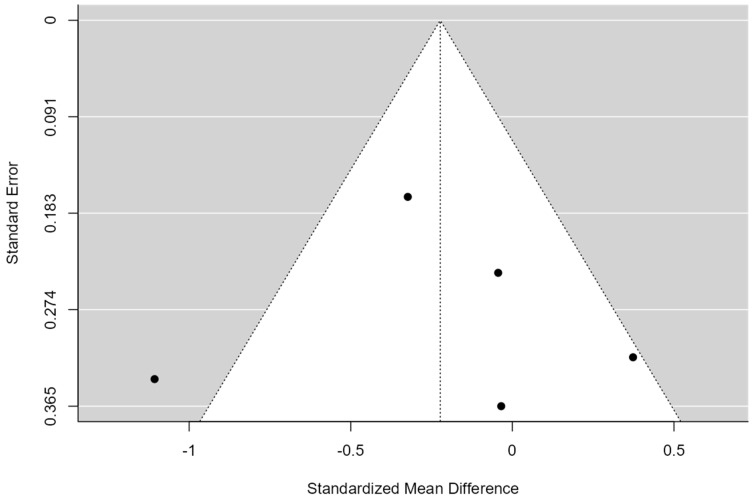
Funnel Plot of manual therapy for shoulder pain and disability (SPADI). The plot is centered on a fixed effect summary estimate, the outer dashed lines indicate the 95% confidence interval of the fixed effect estimates. Symmetry is apparent when all studies are randomly dispersed around the dashed vertical line. Dark circles represent individual studies included in the meta-analyses.

A total of k = 5 studies were included in the analysis. The observed standardized mean differences ranged from −1.10 to 0.37, with the majority of estimates being negative (80%). The estimated average standardized mean difference based on the random-effects model was −0.22 (95% CI: −0.65 to 0.21). Therefore, the average outcome did not differ significantly from zero (z = −1.00, *p* = 0.31). According to the Q-test, the true outcomes appear to be heterogeneous (Q(4) = 11.45, *p* = 0.02, tau^2^ = 0.16, I^2^ = 70.16%). A 95% prediction interval for the true outcomes is provided by −1.13 to 0.68. Hence, although the average outcome is estimated to be negative, in some studies the true outcome may in fact be positive. An examination of the studentized residuals revealed that none of the studies had a value larger than ± 2.57, and hence there was no indication of outliers in the context of this model. According to the Cook’s distances, none of the studies could be considered to be overly influential (see Figure 4). Neither the rank correlation nor the regression test indicated any funnel plot asymmetry (*p* = 1.00 and *p* = 0.94, respectively) (see Figure 5).

#### 3.5.3. Laser Treatment for Shoulder Pain and Disability (SPADI)

**Figure 6:**
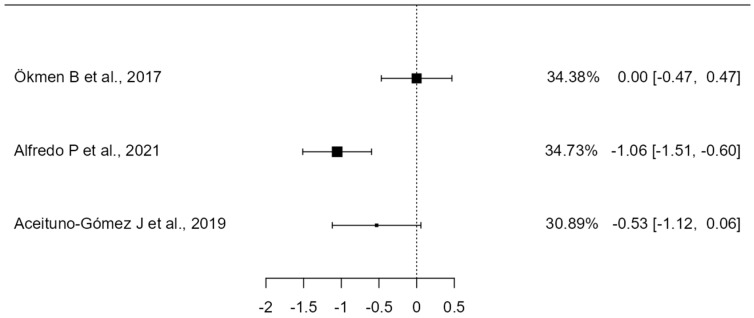
Forest Plot of Laser treatment for shoulder pain and disability (SPADI). The small boxes with the squares represent the point estimate of the effect size and sample size. The lines on either side of the box represent a 95% confidence interval (CI) [31,41,42].

**Figure 7:**
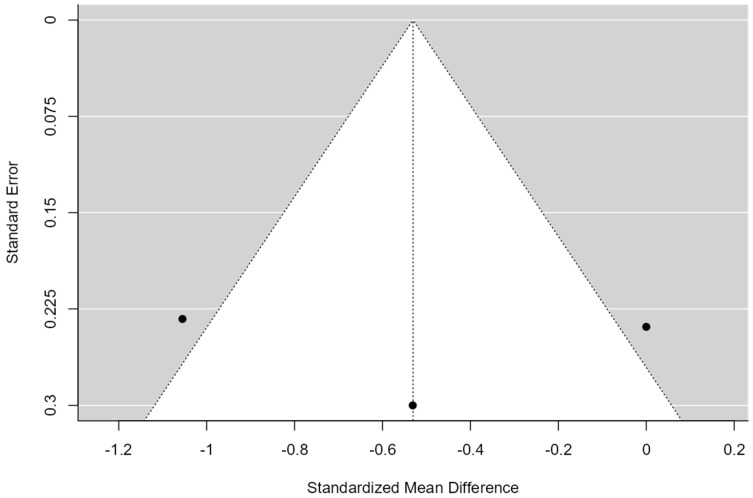
Funnel Plot of laser treatment for shoulder pain and disability (SPADI). The plot is centered on a fixed effect summary estimate, the outer dashed lines indicate the 95% confidence interval of the fixed effect estimates. Symmetry is apparent when all studies are randomly dispersed around the dashed vertical line. Dark circles represent individual studies included in the meta-analyses.

A total of k = 3 studies were incorporated into the analysis. The standardized mean differences observed ranged from −1.05 to 0.00, with the majority (67%) being negative. The estimated average standardized mean difference, derived from the random-effects model, was μ= −0.53 0.53 (95% CI: −1.14 to 0.08). Consequently, the mean outcome did not significantly deviate from zero (z = −1.67, *p* = 0.09). The Q-test suggested heterogeneity among the true outcomes (Q(2) = 9.99, *p* = 0.00, τ^2^ = 0.23, I^2^ = 78.15). A 95% prediction interval for the true outcomes ranged from −1.66 to 0.60. Thus, despite the average outcome being negative, some studies may present a positive true outcome. Examination of the studentized residuals showed no values exceeding ± 2.39, indicating no outliers within this model. Cook’s distances analysis suggested that no study was overly influential (see Figure 6). Furthermore, neither the rank correlation nor the regression test provided evidence of funnel plot asymmetry (*p* = 1.00 and *p* = 0.93, respectively) (see Figure 7).

#### 3.5.4. Exercise Therapy for Shoulder Pain and Disability (SPADI)

**Figure 8:**
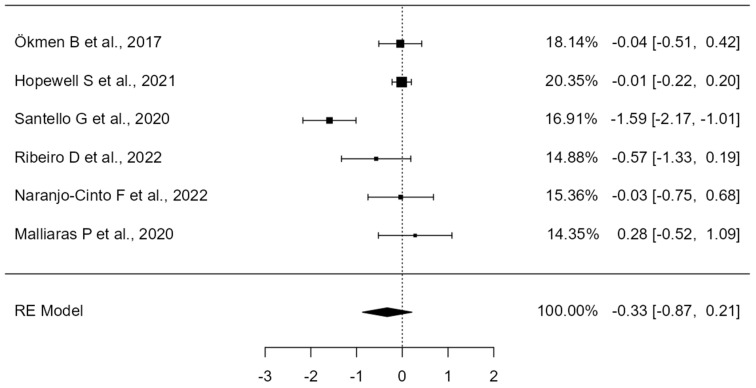
Forest Plot of exercise therapy for shoulder pain and disability (SPADI). The small boxes with the squares represent the point estimate of the effect size and sample size. The lines on either side of the box represent a 95% confidence interval (CI) [31,32,36,38,45,46].

**Figure 9:**
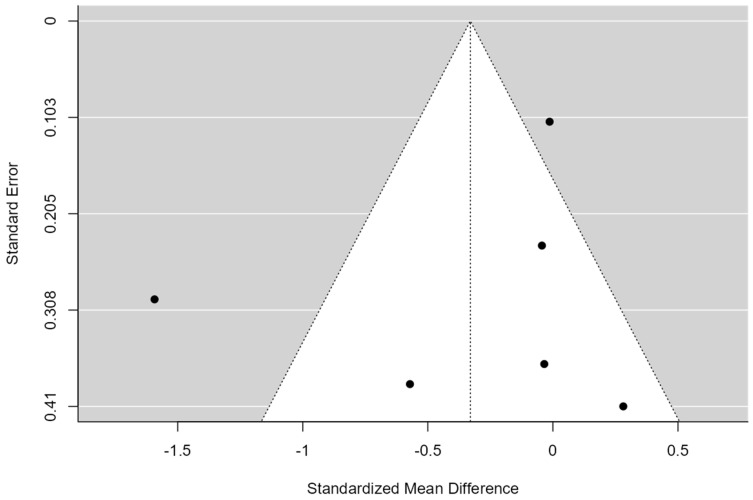
Funnel Plot of exercise therapy for shoulder pain and disability (SPADI). The plot is centered on a fixed effect summary estimate, the outer dashed lines indicate the 95% confidence interval of the fixed effect estimates. Symmetry is apparent when all studies are randomly dispersed around the dashed vertical line. Dark circles represent individual studies included in the meta-analyses.

A total of k=6 studies were included in the analysis. The observed standardized mean differences ranged from −0.37 to 0.21, with the majority of estimates being negative (67%). The estimated average standardized mean difference based on the random-effects model was = −0.0112 (95% CI: −0.24 to 0.22). Therefore, the average outcome did not differ significantly from zero (z = −0.09, *p* = 0.92). According to the Q-test, there was no significant amount of heterogeneity in the true outcomes (Q(5) = 5.47, *p* = 0.36, tau² = 0.02, I² = 28.54%). A 95% prediction interval for the true outcomes is given by −0.40 to 0.37. Hence, although the average outcome is estimated to be negative, in some studies the true outcome may in fact be positive. An examination of the studentized residuals revealed that none of the studies had a value larger than ±2.63 and hence there was no indication of outliers in the context of this model. According to the Cook’s distances, one study (Hopewell S et al., 2021, [32]) could be considered to be overly influential (see Figure 8). The regression test indicated funnel plot asymmetry (*p* = 0.02) but not the rank correlation test (*p* = 0.1361) (see Figure 9).

#### 3.5.5. Exercise Therapy for Shoulder Pain and Disability (DASH)

**Figure 10:**
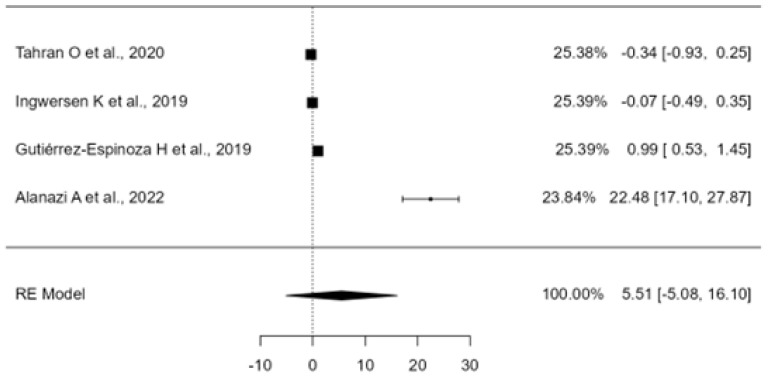
Forest Plot of exercise therapy for shoulder pain and disability (DASH). The small boxes with the squares represent the point estimate of the effect size and sample size. The lines on either side of the box represent a 95% confidence interval (CI) [28,43,44,47].

**Figure 11:**
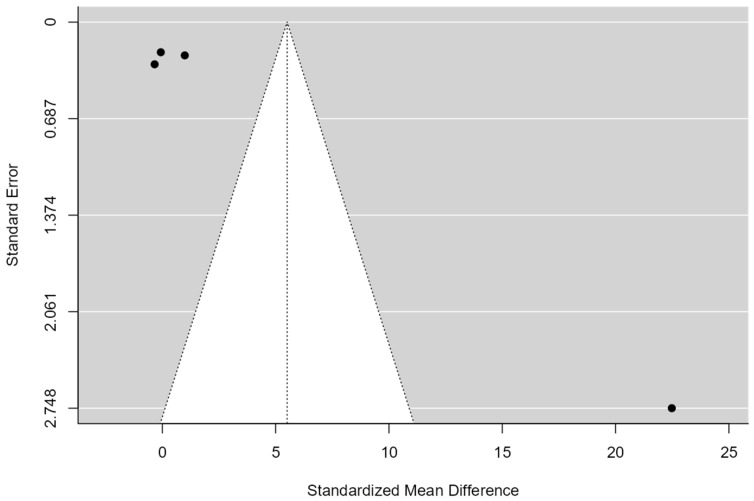
Funnel Plot of exercise therapy for shoulder pain and disability (DASH). The plot is centered on a fixed effect summary estimate, the outer dashed lines indicate the 95% confidence interval of the fixed effect estimates. Symmetry is apparent when all studies are randomly dispersed around the dashed vertical line. Dark circles represent individual studies included in the meta-analyses.

A total of k = 4 studies were incorporated into the analysis. The standardized mean differences observed ranged from −0.34 to 22.48, with half of the estimates being negative. The estimated mean standardized difference, according to the random-effects model, was = 5.50 (95% CI: −5.08 to 16.09). As such, the mean outcome did not significantly deviate from zero (z = 1.01, *p* = 0.30). The Q-test indicated heterogeneity among the true outcomes (Q(3) = 81.14, *p* < 0.00, τ^2^ = 114.93, I^2^ = 99.92). A 95% prediction interval for the true outcomes was −18.02 to 29.03. Consequently, despite the average outcome being positive, some studies may exhibit a negative true outcome. Analysis of the studentized residuals identified one study (Alanazi A et al., 2022 [49]) with a value exceeding ±2.49, suggesting it may be an outlier within this model. Cook’s distances analysis revealed no study to be overly influential(see Figure 10). While the regression test suggested funnel plot asymmetry (*p* < 0.00), the rank correlation test did not (*p* = 0.75) (see Figure 11).

## 4. Discussion

The objective of this study was to determine whether the use of combined physiotherapy treatments adds any benefit to an exercise-only program in patients with shoulder pain and to establish which combined treatment is the most effective. It is difficult to make comparisons between the articles in this review since each study has been conducted in different population samples and the treatments used have varied in each of them. This review shows that the most widely used and the most effective treatment for patients with shoulder pain is therapeutic exercise. However, exercise is applied in combination with other therapies such as laser, self-care advice, pain education, different manual therapy techniques, corticosteroid injections, biofeedback, electropuncture, photobiomodulation, and psychomotor therapy in most articles. Therefore, it is necessary to check the extent to which the addition of other therapies brings added benefit to exercise in the treatment of patients with shoulder pain.

By conducting a comprehensive multimodal meta-analysis (Figure 2), we can gain insight into which combined treatment has yielded the best results in terms of shoulder pain and disability. The greatest reduction in SPADI, with a mean difference of −1.06 (95% CI: −1.51, −0.60) and a study weight of 9.18%, was achieved by Alfredo P et al., 2021 [41]. In this study, in addition to exercise—which included scapular and humeral stabilization exercises and stretching—low-intensity laser therapy was applied for treating 120 patients with SIS.

Aceituno–Gómez J et al., 2019 [42], with a mean difference of −0.53 (95% CI: −1.12, 0.06) and a weight of 7.50%, showed a significant reduction in SPADI through stretching and strengthening exercises combined with high-intensity laser therapy in 46 patients suffering from SIS, although not as pronounced as observed in the study by Alfredo P et al. [41].

Malliaras P et al., 2020 [36] by combining an exercise program with advice and telerehabilitation, achieved a SPADI reduction of −0.38 (95% CI: −1.18, 0.43) with a study weight of 5.33%. This study was conducted on 36 patients with RCRSP.

However, the combination of manual therapy and mobilization along with exercise, as performed by Santello G et al., 2020 [38] in 60 patients with shoulder pain, reported a mean difference of −0.24 (95% CI: −0.74, 0.27) and a weight of 8.50%, with a smaller SPADI reduction compared to the studies by Alfredo P et al. and Aceituno–Gómez J et al. [41] This seems to indicate that adding manual therapy does not provide an additional benefit in reducing shoulder pain and disability.

Although conventional physiotherapy applied by Roddy E et al., 2021 [35] in 256 patients with subacromial pain syndrome, was beneficial, achieving a mean difference of −0.07 (95% CI: −0.42, 0.27) and a weight of 10.73%, its effect was less pronounced than in other studies with more advanced approaches.

On the other hand, Ribeiro D et al., 2022 [45], with a mean difference of −0.23 (95% CI: −0.97, 0.52) and a weight of 5.87%, showed that adding manual therapy to groups diagnosed with shoulder subacromial pain and performing specific shoulder exercises did not result in better outcomes in terms of pain and function. Additionally, this meta-analysis indicated that the results were weaker compared to studies that added other types of techniques to exercise.

Naranjo–Cinto F et al., 2022 [46], in their study, showed a mean difference of −0.14 (95% CI: −0.86, 0.57) and a weight of 6.14%, indicating that adding manual therapy to therapeutic exercise leads to improvements in terms of pain and function. However, these benefits remain less than those obtained with other treatment combinations. In this study, 45 patients with no specific shoulder pain were treated.

Finally, it is necessary to analyze the studies that showed a smaller or no significant reduction in SPADI. Ökmen B et al., 2017 [31], with a mean difference of 0.00 (95% CI: −0.47, 0.47) and a weight of 9.02%, did not show a significant change, suggesting that photobiomodulation therapy and electrotherapy may not be sufficient to effectively reduce SPADI. In this case, 70 patients with chronic shoulder pain were treated.

Land H et al., 2019 [29] reported a mean difference of 0.17 (95% CI: −0.45, 0.79) and a weight of 7.13%, indicating limited effectiveness in combining manual therapy with strengthening exercises in reducing SPADI in patients with SSI.

Dunning J et al., 2021 [30], with a mean difference of 0.10 (95% CI: −0.22, 0.43) and a weight of 11.06%, showed that spinal manipulation therapy along with electrical dry needling and exercise had a lesser impact compared to other therapeutic approaches. In this study, 145 patients with SAPS were treated.

The results shown by Hopewell S et al., 2021 [32], with a mean difference of 0.22 (95% CI: 0.01, 0.43) and a weight of 12.53%, suggest that progressive resistance exercises were effective in improving SPADI, although to a lesser extent than the more advanced approaches that include other therapies for treating 708 patients with rotator cuff disorder.

Finally, Gomes C et al., 2018 [40] reported a mean difference of 0.47 (95% CI: −0.15, 1.10) and a weight of 7.04%, indicating limited effectiveness of using manual therapy and diadynamic currents in reducing SPADI compared to other studies. In this study, 60 patients with SIS were treated.

The results obtained from the various studies have been discussed and grouped according to the type of combined treatment performed to analyze which combination has been most effective. This aims to conduct a less heterogeneous meta-analysis.

### 4.1. Effectiveness of Including Manual Therapy in Terms of Pain and Function (SPADI)

The combination of exercise plus manual therapy has been used by five authors, at least for some of the study groups, obtaining different results that are discussed below.

Land H et al., 2019 [29] showed significant improvements in pain and functionality by combining exercise with manual therapy at the end of the 12-week treatment period. Specific interventions were performed targeting the upper thoracic spine and posterior shoulder region through the application of thoracic spine mobility techniques, massage to the posterior region of the shoulder, and posterior mobilizations of the glenohumeral joint, with neither technique proving superior to the other. As shown in Figure 4, this study demonstrates a reduction in SPADI scores. However, it achieves the least improvement compared to the other articles that include manual therapy.

From the study by Dunning J et al., 2021 [30], we cannot conclude that the inclusion of one type of manual therapy or another in the treatment combined with exercise is more effective. It is observed that more significant improvements are achieved in the group that receives manipulative techniques in lower cervical, cervicothoracic, middle thoracic, and costal joints (1–3), combined with electropuncture than in the group that uses techniques of mobilization of the glenohumeral, acromioclavicular, and periscapular region plus exercise and IFC, which may indicate that the extra benefit of the combined treatment in this study is obtained by the use of the electropuncture. More concise studies looking at the effectiveness of electropuncture applied alone and/or with exercise and/or manual therapy would be necessary in order to draw conclusions. Although Figure 4 shows a moderate shift to the left, suggesting an improvement with results that are only slightly below those obtained by Naranjo–Cinto F et al., 2022 [46], it cannot be deduced from the study by Dunning J et al., 2021 [30] that the addition of one type of manual therapy or another to the exercise treatment program is more effective. More significant improvements are achieved in the group that receives manipulation techniques in the lower cervical, cervicothoracic, mid-thoracic, and costal joints (1–3) when combined with electrical dry needling over the group that uses techniques to mobilize the glenohumeral, acromioclavicular, and periscapular region plus exercise and IFC. This may indicate that the use of electrical dry needling brings extra benefit to the combined treatment. More concise studies looking at the effectiveness of electrical dry needling applied alone and/or with exercise and/or manual therapy would be necessary in order to draw conclusions.

On the other hand, the article by Gomes C et al., 2018 [40] studies the effect of combining the use of diadynamic currents with the application of manual therapy. They showed that all groups improved, but there were significant differences in favour of the group that received both manual therapy and diadynamic currents.

In contrast, Naranjo–Cinto F et al., 2022 [46] found no statistically significant differences between the groups who received manual therapy or placebo manual therapy; thus, it can be concluded that manual therapy adds no extra benefits to exercise treatment as it is shown in Figure 4. Similarly, Ökmen B et al., 2017 [31] found no significant difference between adding manual therapy, photobiomodulation, or radiofrequency to exercise, although both combined treatments were effective. They did not study whether the use of any of these combined treatments was superior to exercise alone. In Figure 4, a slight shift to the left is observed, indicating improvements, but without significant differences between the two methods.

Although most studies show that the addition of manual therapy results in improvements in SPADI scores, the magnitude of these improvements is not always clinically significant. This suggests that manual therapy may not provide substantial additional benefits when added to standard treatments or exercise programs. The variability in manual therapy techniques employed and the therapist’s skill may influence the outcomes, which could explain the differences in the magnitude of improvement observed across different studies.

Common weaknesses in these studies, such as the lack of blinding in some trials, should be considered, as this could introduce biases in the results. Additionally, the heterogeneity in manual therapy techniques and patient populations must be considered. Furthermore, most studies did not find significant differences between real manual therapy and sham interventions, suggesting a potential placebo effect.

Therefore, no strong evidence exists that the addition of manual therapy to therapeutic exercise is crucial, nor is it a differentiating factor in the treatment of patients with shoulder pain. It is crucial to consider the cost-benefit ratio and the accessibility of manual therapies when deciding their inclusion in treatment programs. Future studies should focus on identifying, which subgroups of patients might benefit the most from manual therapy and which specific techniques are the most effective.

### 4.2. Effectiveness of Including Laser Treatment in Terms of Pain and Function (SPADI)

Three authors studied the effectiveness of adding laser application to exercise in the treatment of patients with shoulder pain using different intensity parameters.

Alfredo P et al., 2021 [41] studied the effectiveness of using low-intensity lasers and concluded that it did not bring any benefit compared to exercise alone.

Aceituno–Gómez J et al., 2019 [42] compared the effectiveness of applying low-intensity lasers together with exercise versus placebo laser plus exercise and obtained a similar improvement in both groups. Ökmen B et al., 2017 [31] found no significant differences adding photobiomodulation or radiofrequency to exercise.

Although all three articles indicate improvements in SPADI scores with the addition of laser therapy, the clinical significance of these improvements varies. Alfredo P et al., 2021 [41] shows the most clinically significant results, suggesting that laser therapy can greatly enhance the effectiveness of exercise programs for shoulder pain. However, the improvements in the other articles, while positive, may not always be sufficient to justify the additional resources required for laser therapy.

The results obtained by including laser therapy should be interpreted with caution, as the differences in outcomes between various studies may be influenced by the practitioner’s skill and the parameters employed. This variability underscores the necessity of developing and analyzing standardized protocols to ensure consistent results. Future studies should aim to identify the most effective laser therapy parameters and evaluate the impact of practitioner experience on patient outcomes. Additionally, the possibility of a placebo effect should be considered, as some studies do not suggest significant differences between actual laser therapy and sham interventions. The lack of blinding in certain trials could introduce biases and affect the results. It is essential to design rigorous studies with appropriate blinding to obtain reliable and unbiased evidence.

Future research should focus on identifying subgroups of patients who may benefit the most from laser therapy and determining which specific parameters are most effective.

Therefore, the results obtained by different authors seem to indicate that the use of low- or high-intensity laser or photobiomodulation combined with exercise does not provide any additional benefit in the treatment of patients with shoulder pain, although it generally helps to reduce pain and disability.

### 4.3. Effectiveness of Exercise Modalities in Terms of Pain and Function (SPADI)

When analyzing which therapeutic exercise protocols were most effective in reducing shoulder pain and disability, we found that Santello G et al., 2020 [38] achieved the best results by implementing a home exercise program that included self-stretching, joint mobility, and strengthening exercises. The high adherence to the program and the inclusion of strengthening and stretching techniques contributed to the positive outcomes. The educational and emotional and cognitive support provided in this study appear to be key in the shoulder treatment approach through exercise. Ribeiro D et al., 2022 [45] was the second author who achieved better results by applying standardized or specific exercises mixed with manual therapy. However, it is noteworthy that, although this author achieved an overall reduction in SPADI values, no significant differences were found between groups, suggesting that the application of specific exercise proposed in the study to restore movement patterns and normalize muscle strength did not provide a relevant benefit. On the other hand, Ökmen B et al., 2017 [31], in their application of exercise combined with photobiomodulation or radiofrequency, also showed general improvements in SPADI values, although there were no significant differences between the two groups. Therefore, in this case, we can think that the success of this intervention might be due to the type of exercises performed, which consisted of Codman exercises, stretching and strengthening exercises, and exercises to gain range of motion.

In contrast, Naranjo–Cinto F et al., 2022 [46] achieved a reduction in SPADI values, but these were not as significant compared to the studies by Santello G et al., 2020 [38], Ribeiro D et al., 2022 [45], and Ökmen B et al., 2017 [31]. This lower efficacy could be due to the type of exercise performed, which was more general, and the number of sessions, which was also fewer than in the studies by Santello G et al., 2020 [38], Ribeiro D et al., 2022 [45], and Ökmen B et al., 2017 [31].

Lastly, Hopewell S et al., 2021 [32] and Malliaras P et al., 2020 [36] achieved more modest improvements compared to the other articles. It is worth noting that Malliaras P et al., 2020 [36] showed better results when combining exercise with education on pain mechanisms and causes of the pathology, and with the combination of a telerehabilitation program consisting of self-management exercises and education, although the results were not significant.

Therefore, advice, self-management techniques, pain education, and understanding the pathology seem to have positive effects on the recovery of patients with shoulder pain, but they do not add a significant benefit over exercise programs. However, it seems reasonable to think that the inclusion of strengthening exercises in exercise programs can offer additional significant benefits in the treatment of shoulder pain and disability. Individualizing the treatment and ensuring proper progression are key to maximizing results. Future studies should focus on standardizing exercise protocols and conducting randomized controlled trials with adequate sample sizes and blinding to reduce biases and improve the robustness of the findings.

### 4.4. Effectiveness of Exercise Modalities in Terms of Disabilities of the Arm, Shoulder, and Hand (DASH)

Tahran O et al., 2020 [28] achieved the best results in reducing DASH scores by including conventional physiotherapy with “modified cross-body stretch” (MCS) and “modified sleep stretch” (MSS) exercises. However, there were no significant differences between the two types of stretches, suggesting that the overall improvements might be attributed to the exercises performed in both groups, which consisted of strengthening exercises for the scapular, rotator cuff, and deltoid musculature, as well as stretching of the trapezius muscle. On the other hand, Ingwersen K et al., 2019 [43] obtained the second-best results but did not achieve significant improvements by adding psychomotor therapy to the exercise regimen. Therefore, their results are likely due to the employment of strengthening and stabilization exercises focused on the rotator cuff and thoracic muscles. Gutiérrez–Espinoza H et al., 2019 [44] obtained modest results by adding pectoralis minor stretches to the specific exercise program, with this inclusion not providing significant additional benefits. Lastly, Alanazi A et al., 2022 [47] showed the poorest results, although they demonstrated that adding handgrip strengthening exercises provided an extra benefit in improving shoulder function.

Different authors have proposed and studied many combined treatments to correctly manage patients with shoulder pain, as can be seen in the aforementioned studies. As we have discussed earlier, exercise serves as the foundational treatment most commonly employed. Analyzing all combinations of exercise with other types of therapies reveals that some of the best outcomes were observed in studies that included laser therapy, advice, telerehabilitation, and to a lesser extent, manual therapy. However, when examining these studies individually, we find that most therapies added to exercise did not yield significant differences on their own. Therefore, we can conclude that these additional therapies do not provide extra benefits in terms of shoulder function, pain, and disability.

In contrast, some combinations have proven to be beneficial. For instance, adding advice and education to exercise regimens resulted in higher adherence to treatment, which can be crucial. Similarly, it seems important that exercises focus on strengthening the shoulder and scapular region musculature.

Despite this, most studies use different diagnostic labels even when measuring similar variables and end up applying similar treatments, primarily exercise, consistently achieving good results to some extent. This variability makes it difficult to compare different studies due to the heterogeneity of the samples, complicating the determination of the most effective treatment for shoulder pain. Additionally, the duration of treatments investigated often varies, further complicating comparisons.

Several reflections may be necessary. Firstly, the choice of treatment should correspond to the patient’s functional characteristics at the time of seeking treatment. This necessitates research that establishes treatment groups with similar samples concerning aspects such as range of motion (ROM), function, and muscle strength. In other words, protocols should be developed to determine the type and progression of exercises each patient should perform based on their initial functional characteristics and how these evolve throughout the treatment. Therefore, research needs to be conducted so that the relationships between the main characteristics of each patient and the treatment that offers the best improvement for these characteristics are identified.

For example, it stands to reason that treatments that focus on aspects such as pain education, self-management techniques, and advice, in addition to exercise, may be more indicated for patients with high levels of kinesophobia, or that combined treatments that focus on the application of exercise alone or more manual therapy may be indicated for patients who have a decrease in ROM and/or have muscle strength deficits in the entire shoulder region. Likewise, the state of the muscle would determine the different exercise protocols established; these protocols would take into account the different load progression, the range of exercises, and whether biofeedback was included or not.

Therefore, future lines of research that focus on studying, which combined treatments, are the most appropriate and beneficial in patients with shoulder pain, based on variables such as kinesophobia, disability, ROM, functionality, and muscle strength, in the short and long term, are needed.

### 4.5. Methodological Quality

The methodological quality of the included studies varied, with most scoring “good” on the PEDro scale. Common weaknesses included a lack of blinding and heterogeneity in intervention protocols. These factors could introduce biases and affect the reliability of the results. Future studies should aim for higher methodological rigor, including better blinding and standardized treatment protocols.

### 4.6. Strengths and Limitations

The study presents several strengths and limitations that should be considered. Among the strengths, the comprehensive review and meta-analysis of recent studies provide an up-to-date and thorough understanding of the effectiveness of combined treatments for shoulder pain. Additionally, the inclusion of a wide range of combined therapies allows for a comparative evaluation of different therapeutic approaches. The use of standardized outcome measures such as SPADI and DASH ensures the comparability and consistency of the results obtained. However, the study also has some limitations. One of the main limitations is the heterogeneity in intervention protocols and patient populations, which may hinder the generalizability of the results. Furthermore, the methodological quality of the included studies varies, with some studies exhibiting weaknesses in aspects such as blinding, which could introduce biases and affect the reliability of the results. The lack of blinding in some studies might have influenced the reported outcomes. These variabilities in methodological quality and treatment protocols underscore the need for future studies with greater methodological rigor, including better blinding practices and standardized treatment protocols. Finally, more databases with longer search periods and grey literature could have been used. With these strengths and limitations, it is crucial for future research to focus on standardizing treatment protocols and conducting randomized controlled trials with adequate sample sizes and appropriate blinding to reduce biases and improve the robustness of the findings.

## 5. Conclusions

This systematic review and meta-analysis evaluated the effectiveness of combined physiotherapy treatments compared to exercise-only programs in patients with shoulder pain. The results demonstrated that therapeutic exercise is fundamental for treating shoulder pain, with additional benefits observed when combined with other therapies such as low-intensity laser therapy and manual therapy. Low-intensity laser therapy, in particular, showed a significant reduction in SPADI scores (mean difference of −1.06; 95% CI: −1.51 to −0.60). The methodological quality of the included studies varied, with the majority scoring “good” on the PEDro scale. Common issues included a lack of blinding (present in over half of the studies), heterogeneity in treatment protocols, and variability in sample sizes. These factors could introduce biases and affect the reliability of the results. Notably, only a few studies achieved “excellent” methodological quality, and several studies did not use intention-to-treat analysis, which may have impacted the findings. The combination of exercise with other therapies showed moderate benefits, although the magnitude of these benefits varied across studies. The variability in the quality and execution of these studies underscores the need for future research with greater methodological rigor. This study highlights the need to standardize treatment protocols and identify specific patient subgroups that may benefit the most from combined therapeutic approaches. Future research should focus on conducting randomized controlled trials with adequate sample sizes, appropriate blinding, and the use of intention-to-treat analysis to ensure robust and unbiased results. Additionally, long-term studies are necessary to evaluate the sustainability of treatment benefits and the cost-effectiveness of combined therapies. In summary, while combined therapies can offer additional benefits, therapeutic exercise remains the most effective intervention for shoulder pain. Current evidence supports the inclusion of additional therapies based on individual patient needs and underscores the importance of continued research to optimize treatment approaches.

## Figures and Tables

**Table 1:** Methodological quality evaluation using the PEDro scale.

Authors	Item											Total	Result
	1 *	2	3	4	5	6	7	8	9	10	11		
Tahran O et al., 2020 [28]	1	1	0	1	0	0	1	1	1	1	1	7/10	Good
Land H et al., 2019 [29]	1	1	1	1	0	0	1	1	0	1	1	7/10	Good
Dunning J et al., 2021 [30]	1	1	1	1	0	0	1	1	1	1	1	8/10	Good
Ökmen B et al., 2017 [31]	1	1	0	1	1	0	1	1	0	1	1	7/10	Good
Hopewell S et al., 2021 [32]	1	1	0	1	0	0	0	1	1	1	1	6/10	Good
Eliason A et al., 2021 [33]	1	1	1	1	0	0	1	1	1	1	1	8/10	Good
Menek B et al., 2019 [34]	1	1	1	1	0	0	1	1	0	1	1	7/10	Good
Roddy E et al., 2021 [35]	1	1	0	1	0	0	1	1	1	1	1	7/10	Good
Malliaras P et al., 2020 [36]	1	1	0	1	0	0	1	1	0	0	1	5/10	Fair
De Oliveira A et al., 2022 [37]	1	1	1	1	0	0	1	1	1	1	1	8/10	Good
Santello G et al., 2020 [38]	1	1	1	1	0	0	1	1	1	1	1	8/10	Good
Moslehi M et al., 2021 [39]	1	1	1	1	0	0	1	1	0	1	1	7/10	Good
Gomes C et al., 2018 [40]	1	1	1	1	0	0	1	1	1	1	1	8/10	Good
Alfredo P et al., 2021 [41]	1	1	1	1	0	0	1	1	0	1	1	7/10	Good
Aceituno–Gómez J et al., 2019 [42]	1	1	0	0	1	0	1	1	0	1	1	6/10	Good
Ingwersen K et al., 2019 [43]	1	1	0	1	0	0	1	1	1	1	1	7/10	Good
Gutiérrez–Espinoza H et al., 2019 [44]	1	1	1	1	0	0	1	1	1	1	1	8/10	Good
Ribeiro D et al., 2022 [45]	1	1	1	1	1	0	1	1	1	1	1	9/10	Excellent
Naranjo–Cinto F et al., 2022 [46]	1	1	1	1	1	0	1	1	1	0	1	8/10	Good
Alanazi A et al., 2022 [47]	1	1	0	1	0	0	1	1	0	1	1	6/10	Good

Abbreviations 0. The criterion is not met by the article. 1. The criterion is met by the article. 1*. Specification of selection criteria. 2. Random assignment of subjects to groups. 3. Concealment of the assignment. 4. In regard to the predicative indicators of highest importance, similarity was seen between groups at baseline. 5. Blinding of all participants. 6. The administration of the therapy was conducted by blinded therapists. 7. Each evaluator measuring one or more outcomes was blinded. 8. Of the initial subjects assigned to a group, a minimum of 85% of them had measurements of at least one of the crucial outcomes. 9. Each participant who was treated or assigned as a control had results shown; when this was not possible, no less than one crucial outcome was critiqued by “intention to treat”. 10. As regards to the results for statistical comparison between groups, no less than one crucial outcome was reported. 11. No less than one crucial outcome was provided for variability and point measurements. * PEDro scale criterion for non-summation.

**Table 2:** Characteristics of Included Studies.

Authors	Participants	Treatment	Outcome Measures	Main Results
Tahran O et al., 2020 [28]	67 patients with SIS and GIRD. Mean age: 52.94 ± 11.05 years. G1 (n = 22): treatment program + MCS exercise. G2 (n = 23): treatment program + MSS exercise. G3 (n = 23): control group (treatment program).	Treatment program: - 20 min of heat application; 20 min of high-frequency (50–100 Hz), low-intensity, small pulse width (50–200 μs) conventional transcutaneous electrical nerve stimulation; and 5 min of 1 MHz, 1.5 W/cm^2^ continuous ultrasound. - Wand exercises, posture exercises, and Codman exercises (10 repetitions of each) - Upper trapezius stretching (five repetitions). - Strength training for the scapular stabilizers, rotator cuff, and deltoid muscles using an elastic band (three sets of 10 repetitions, with 1 min rest between sets). MCS and MSS program: - One set of 5 repetitions of a 30 s stretch was performed. Exercises: once a day, every day, for 4 weeks. Treatment: 20 sessions five times/week.	Measurements were taken on the 1st day and the day after treatment was completed. VAS: pain at rest and activity. CMS: shoulder function. QuickDASH: disability level. Bubble inclinometer: shoulder mobility (PST, IR ROM, and ER ROM).	All groups improved pain, function, and ROM (*p* < 0.05). Groups 1 and 2 obtained better results than Group 3 (*p* < 0.05). No significant difference between G1 and G2 was found (*p* > 0.05).
Land H et al., 2019 [29]	60 patients with SSI. Mean age: G1 = 51 ± 4.4; G2 = 51 ± 5.4; G3 = 51 ± 6.0 G1 (n = 20): upper thoracic spine mobilization (passive) + home exercise. G2 (n = 20): posterior shoulder massage, passive mobilization + stretching. G3 (n = 20): active control (ultrasound).	Upper thoracic intervention (G1): - Thoracic transverse and costovertebral mobilization on the side of painful shoulder (T1-T6) 20 min, nine sessions for 6 weeks. - Home exercise: passive thoracic extension, 5 min, twice a day up to 12 weeks. Posterior shoulder intervention (G2): - 15 min of massage, focusing along length of infraspinatus and teres minor nine sessions (6 weeks). - Anteroposterior glenohumeral mobilization for 2 min, nine sessions (6 weeks). - Passive cross-adduction stretch, 20 s, 2 times/day for 12 weeks. Ultrasound (G3): 1 MHz 50% pulsed 0.5 w/cm^2^ for 8 min at the subacromial area nine sessions for 6 weeks.	NPRS SPADI Passive IR Posterior shoulder Thoracic resting Active thoracic range. Measurements were taken at baseline at 3 weeks at 6 weeks. at 9 weeks. at 12 weeks.	Both combined treatments of thoracic spine mobilization + exercise or posterior shoulder massage and mobilization + exercises have improved function and passive IR range in patients with SSI.
Dunning J et al., 2021 [30]	145 patients with SAPS. Mean age: G1 = 46.2 ± 15.6; G2 = 47.8 ± 15.8 G1 (n = 73): TMEDN group (spinal manipulation + electrical dry needling) G2 (n = 72): NTMEX group (peripheral mobilization + exercise + IFC	Twelve treatment sessions: two per week for 6 weeks. TMEDN group (G1): - Thrust manipulation: lower cervical (C4–C6), cervicothoracic (C7–T3), midthoracic (T4–T9), and upper-rib articulations (1–3). - Electrical dry needling (20 min): eight points in subacromial and scapular regions and six needles in the upper thoracic paraspinal, peri-scapular, and glenohumeral regions. Low-frequency (2 Hz) moderate-pulse-duration (250 microseconds), biphasic continuous waveform at a “moderate” intensity. NTMEX (G2). - Nonthrust mobilization: glenohumeral joint, acromioclavicular joint, and peri-scapular-region. - Exercise: three sets of 10 repetitions with bands - Stretching: three sets of 30 s 8–15 v minutes of soft tissue mobilization in the posterior and anterolateral shoulder region. - 15–20 min of IFC around the subacromial space region; 15-to-120 Hz and a “strong but comfortable tingling” intensity.	Measurements taken at baseline, 2 weeks and 4 weeks: SPADI NPRS Measurements, taken at 2 weeks, 4 weeks, and 3 months: GROC Medication intake was assessed at baseline and 3 months after first treatment session.	Both groups improved shoulder symptoms and function. TMEDN group obtained greater results, with higher changes for SPADI than NTMEX group at 4 weeks and at 3 months (−26.9 vs. −16.3 and −35.1 vs. −17.1). (*p* < 0.001). Results for NPRS were better for TMEDN group at 4 weeks and at 3 months (changes: −3.2 vs. −2.0 and −4.0 vs. −1.9). (*p* < 0.001) More patients from TMEDN group ceased the pain medication pain at 3 months (74% vs. 32%). GROC results were better for G1. The effect of duration of symptoms was similar in both groups.
Ökmen B et al., 2017 [31]	70 patients with chronic shoulder pain. Mean age: G1 = 52.3 ± 8.7; G2 = 52.5 ± 8.6 G1 (n = 35): Group H G2 (n = 35): Group P	14 days treatment over 2 weeks. Group H: exercise + PBMT with high-powered device). PBMT (2 phases on the most painful area): Phase I: 2 sessions at 48 h intervals. Pulsed mode at 1064 nm wavelength and 8 W power for 250 s with a frequency of 25 Hz and pulse duration time less than 150 ms. Phase II: continuous mode at 1064 nm wavelength and 7 W power for 357 s. Group P: exercise + SSN-pulsed RF therapy). SSN-pulsed RF therapy: 45 V, 200 ms, 42 °C for 240 s (4 min). Exercise protocol: ROM exercises, Codman’s exercises, and stretching and strengthening exercises. Five repetitions of each, twice a day.	Measurements taken at pretreatment (PRT) and posttreatment (PST) at 0, 1, 3, and 6 months. SPADI VAS NHP scoring system	Both groups showed statically significant differences for SPADI, VAS and NHP scoring system (*p* < 0.05) at all measurement times. There were no statically significant differences between groups (*p* > 0.05) for all outcome measurement times.
Hopewell S et al., 2021 [32]	708 patients with rotator cuff disorder. Mean age: G1 = 55.9 ± 13.1; G2 = 56.5 ± 12.4; G3 = 54.6 ± 13.7; G4 = 58.8 ± 13.2. G1 (n = 174): Progressive exercise. G2 (n = 174): best practice advice. G3 (n = 182): corticosteroid injection + progressive exercise. G4 (n = 178): corticosteroid injection + best practice advice.	The corticosteroid injection was either methylprednisolone acetate (≤40 mg) or triamcinolone acetonide (≤40 mg). The local anaesthetic was either 1.0% lidocaine (≤5 mL) or 0.5% bupivacaine hydrochloride (≤10 mL). Exercise protocol: five sessions/week for 16 weeks. Resisted external rotation, flexion, and abduction of the shoulder exercises with resistance bands. Best practice advice intervention: a single individual face-to-face session with a physiotherapist for 60 min.	Measurements taken at baseline, 8 weeks, 6 months, and 12 months after randomisation: SPADI SPADI pain subscale SPADI function subscale. Fear-avoidance belief questionnaire. Pain self-efficacy questionnaire. Insomnia severity index. Return to desired activities.	All groups obtained similar SPADI results at 12 months. At 8 weeks corticosteroid injection improves shoulder pain *p* ≤ 0.0001. Progressive exercise was not superior to a best practice advice session with a physiotherapist in improving shoulder pain and function (*p* ≥ 0.005).
Eliason A et al., 2021 [33]	120 patients with SPS. Mean age: G1 = 43.2 ± 9.8; G2 = 45.5 ± 8.3; G3 = 46.0 ± 10.2. G1 (n = 29) intervention group 1: joint mobilization + guided exercises. G2 (n = 52) intervention group 2: guided exercises. G3 (n = 39) control group: no treatment.	Joint mobilizations (lateral, dorsal, and ventral mobilization of the head of the humerus): eight sessions for 6 weeks (1–2/week). Each mobilization was repeated three times and held for 30 s. Guided exercise training: 20 sessions for 12 weeks. Exercise program: retraction of the scapulae, adduction, outward rotation with fixated elbow, abduction, depression of the shoulder, stretching of the upper trapezius and pectorals, and pendulum. Using dumbbells or resistance bands. Three sets of 10 repetitions. Pain between 10–40 on VAS is allowed during exercises.	Measurements taken at baseline, 6 weeks, 12 weeks, and 6 months: C-M VAS AROM	All groups obtained better C-M scores and were higher in G1 and G2 at 12 weeks and 6 months. Significant increase in AROM was found in all groups (*p* < 0.05). No differences between groups were found for AROM. G1 obtained better results for decreased pain in AROM at 6 and 12 weeks (*p* < 0.05). Add-on joint mobilization is more effective than exercise alone or no treatment for decreasing pain in the short term.
Menek B et al., 2019 [34]	30 patients with Rotator cuff syndrome. Mean age: G1 = 51.73 ± 6.64; G2 = 50.26 ± 4.28. G1 (n = 15) Mulligan group (exercise program + Mulligan mobilizations + ultrasound + TENS). G2 (n = 15) Control group (traditional physiotherapy + exercise program + ultrasound + TENS).	6 weeks of treatment. Traditional physiotherapy: stretching exercises, cold pack, TENS, finger staircase, Codman, and wand exercises. Ultrasound: 1.5 MHz for 6 min. TENS: 100 Hz for 20 min. Exercise program: 5 days/week for 6 weeks, once a day. Wand exercises, shoulder capsule stretching, Codman exercises, shoulder flexion, abduction, extension, and external and internal rotation strengthening exercises. Three sets of 10 repetitions. Mulligan mobilizations: active mobilizations of the humeral head using the motion with mobilization technique (pain-free). Flexion, abduction, external and internal rotation mobilizations. Three sets of 10 repetitions for 20 min with 30 s of rest between each set.	Measurements taken at baseline and posttreatment: VAS resting. VAS activity. ROM DASH SF-36 questionnaire.	All groups showed good results posttreatment but G1 (Mulligan group) obtained better results than G2 in ROM, VAS, and DASH (*p* < 0.05). SF-36 questionnaire results improved in both groups.
Roddy E et al., 2021 [35]	256 patients with subacromial pain syndrome. Mean age: Overall = 53.8 ± 10.2; G1 = 55.6 ± 10.5; G2 = 54.8 ± 10.0; G3 = 51.9 ± 10.7; G4 = 53.0 ± 9.5 G1 (n = 64): US-guided corticosteroid injection + physiotherapist-led exercise. G2 (n = 64): US-guided corticosteroid injection + leaflet. G3 (n = 64): unguided injection + physiotherapist-led exercise. G4 (n = 64): unguided injection + leaflet.	Physiotherapist-led exercise: individualised, supervised and progressive exercise, 6–8 sessions over 12–16 weeks. Scapular stability exercise without resistance, isometrics and stretching exercises with scapular control in pain-free range and resistance exercises to encourage rotator cuff muscle strengthening. Advice and exercise leaflet: Information about shoulder anatomy and SAPS, simple self-help massages for analgesia, cold packs and 6 specific strengthening and range of motion exercises (2–3 times/day, without instructions for progression or individualisation). Corticosteroid injection: premixed solution of methylprednisolone 40 mg and 1 mL 1% lidocaine was injected into the bursa.	Measurements taken at baseline, 6 weeks, 6 months, and 12 months: SPADI total score. SPADI pain and function subscores. Pain severity today (NRS). Short Form-12. Fear avoidance (Tampa scale for kinesiophobia). Pain self-efficacy.	There were no significant differences between the US-guided injection group and the unguided injection group. The physiotherpist-led exercise groups obtained better results than the leaflet groups. Total SPADI score had greater improvement at 6 months (*p* < 0.05).
Malliaras P et al., 2020 [36]	36 patients with RCRSP. Mean age: G1 = 53.7 ± 11.5; G2 = 51.3 ± 13.7; G3 = 56.6 ± 11.0 G1 (n = 12): Advice only. G2 (n = 12): Advice + recommended care. G3 (n = 12): Advice + recommended care + telerehabilitation.	Duration of the treatment: 12 weeks. Advice only: patients received education about the rotator cuff muscles and risk factors and advice about modifying general and work-related activities. Patients were advised to carry out their activities with acceptable pain. Advice with recommended care: patients received education about the pain mechanisms and causes of RCRSP (according to the evidence-based principles of self-management and cognitive behavioral therapy) + exercise. Telerehabilitation: Two sessions of 60 min in which a physiotherapist provided education about RCRSP, exercises, and self-management. The other sessions (30 min) discussed beliefs about pain and pathology, expectations, etc. Exercises: three times/week for 12 weeks. Three sets of 15 repetitions and 4 s/cycle for isotonic exercises. The weight must be adapted according to the ability to do more or fewer repetitions. Shoulder elevation in standing position from 10 to 150 degrees, and external rotation in side-lying position, full range.	Measurements taken at baseline, 6 weeks, and 12 weeks: SPADI. Patient Global Rating of Change 11-point Likets scale. VAS Kinesiophobia (TSK) PCS PSEQ	All groups showed improvements in the measured variables. Groups 2 and 3 obtained better results, but the differences between these two groups are not shown.
De Oliveira A et al., 2022 [37]	24 patients with SPS. Mean age:46.2 ± 2 G1 (n = 12): Experimental biofeedback group (exercise + biofeedback). G2 (n = 12): exercise group (therapeutic exercise).	Treatment: 2 days/week for 8 weeks. 40 min per session. Exercises: medial and lateral rotation movements of the shoulder + scapular retraction. Scapular retraction + extension movement with elastic band. The load was adapted using different bands. “Push-up plus” exercise. Biofeedback: EMG-biofeedback in the UT, MT, LT, and SA muscles was used while the patient performed the exercises.	Measurements taken at baseline, 4 weeks, 8 weeks, and 12 weeks: NPRS DASH	Both groups improved. At 8 weeks G1 showed better results for NPRS than G2 (*p* = 0.01). There were no significant differences between groups for the rest of the variables.
Santello G et al., 2020 [38]	60 patients with shoulder pain. Mean age: G1 = 53 ± 12; G2 = 54 ± 15 G1 (n = 30): Intervention group. G2 (n = 30): Control group.	G1-intervention group: Home-based exercise program. 3 times/week for 2 months. Exercises should be done free of pain. In the first session, patients received a booklet with descriptions of the exercises and instructions from the therapist. Exercises: Self-stretching of the trapezius, minor pectoralis, and posterior and inferior structures of the shoulder (3 sets of 30 s). Joint mobility: shoulder abduction, scapular retraction and depression, and shoulder elevation (three sets of 5–10 repetitions). Strengthening: internal and external rotator and abductor muscles and SA (three sets of 5–10 repetitions). G2 control group: patients received an explanation about their shoulder pain and advice on self-care (neck self-massage, use of ice, activities to avoid, etc.)	Measurements taken at baseline and after 2 months: SPADI NPRS CPSS SF-36	All variables improved more in G1 compared to G2 (*p* < 0.05), except quality of life, which improved in both groups.
Moslehi M et al., 2021 [39]	75 patients with SIS. Mean age: G1 = 38.3 ± 7.4; G2 = 37.5 ± 8.1; G3 = 38.2 ± 4.1 G1 (n = 25): Scapular-focused treatment with feedback. G2 (n = 25): Scapular-focused treatment. G3 (n = 25): Control group.	Scapular-focused treatment: 8 weeks of exercises including isometric stretching, intrinsic and eccentric isotonic exercises, shoulder position training, rotator cuff muscle strength and flexibility. Feedback intervention: during each exercise patients were guided by the therapist using tactile and verbal feedback in the scapula and pre-scapular muscles.	Measurements taken at baseline and 8 weeks: VAS DASH	For pain and DASH, significant differences were obtained between groups, with group 1(the group that received feedback) obtaining the greatest improvement. Pain: G1–G2 *p* = 0.04; G1–G3 *p* = 0.01. DASH: G1–G2 *p* = 0.03; G1–G3 *p* = 0.01.
Gomes C et al., 2018 [40]	60 patients with SIS Mean age: G1 = 40.45 ± 6.64; G2 = 38.45 ± 4.95; G3 = 35.55 ± 5.85 G1 (n = 20): MTDD group (manual therapy and diadynamic currents) G2 (n = 20): MT group (manual therapy). G3 (n = 20): DD group (diadynamic currents).	16 treatment sessions for 8 weeks. Manual therapy: lateral inclination of the cervical spine toward the affected shoulder combined with elevation of the shoulder and with manual contact on myofascial trigger points (3 sets of 90 s) + ischemic compression over myofascial trigger points (3 sets of 90 s). Diadynamic currents: positioned over the myofascial trigger point in the upper trapezius (negative electrode) and between the scapula (positive electrode). Fixed biphasic modality (4 min) + 4 min of long periods + 4 min of short periods. Intensity: at sensory threshold (Modalities 1 and 2) and at motor threshold (Modality 3).	Measurements taken at baseline and post intervention: SPADI NRPS	All groups obtained statistically significant results for the measured variables (*p* < 0.05). The group that received manual therapy + diadynamic currents obtained better results than the groups that received a single treatment, with the difference being statistically significant for the SPADI and for the NRPS (*p* < 0.05).
Alfredo P et al., 2021 [41]	120 patients with SIS. Mean age: G1 = 51.9 ± 8.7; G2 = 56.0 ± 10.4; G3 = 54.2 ± 7.1 G1 (n = 42): Low-level laser therapy + exercises. G2 (n = 42): Exercises only. G3 (n = 36): Low-laser therapy only.	Three times/week for 8 weeks. Low-laser therapy: 3 J of energy per point in three insertion points each in the supraspinatus muscle tendon, on the subacromial bursa, and along the bicipital groove. Wavelength of 904 nm, frequency of 700 Hz, average power of 60 mW, peak pulse power of 20 W, and 50 s of irradiation per point (area, 0.5 cm^2^). Exercises for scapular pivot, scapula stabilizer, and humeral propellant muscle groups: Isotonic muscle strengthening, three sets of 15 repetitions. Isometric exercises, 10 sets of 10 s. Stretching of the trapezius, pectoralis minor muscles, posterior and inferior shoulder structures. Patients in G1 received the low-laser therapy before the exercises.	Measurements taken at baseline, 2 months, and 3 months follow-up: PAIN DLA PAIN REST SPADI	All groups improved their results. The group that combined laser therapy with exercise obtained statistically significant differences in the between groups comparison for all variables (*p* ≤ 0.05).
Aceituno-Gómez J et al., 2019 [42]	46 patients with SIS. Mean age: G1 = 56.7 ± 8.9; G2 = 61.3 ± 8.9 G1 (n = 23): High-intensity laser therapy + exercise (experimental group). G2 (n = 23): Sham-laser + exercise (sham-controller group).	A total of 15 sessions, five sessions/week for 3 weeks. Exercise protocol: stretching and strengthening exercises. Laser treatment: wavelength of 1064 nm with 15 W maximum power output. 2 phases: (i) applying a power of 12 W at a frequency of 50 Hz and a 20% work cycle, during which 50 J/cm^2^ were administered; and (ii), applying a power of 15 W in burst mode (10 pulses for 900 ms per train), during which 250 J/cm^2^. Sham-laser treatment group received the same procedure with the guide light on the device switched on but at 0 W output.	Measurements taken at baseline, 1 month, and 3 months: VAS SPADI CMS QuickDASH scale Pressure Pain Threshold	Both groups obtained statistically significant improvements in pain and function (*p* ≤ 0.05). The group that received laser therapy versus sham-laser treatment did not obtain significant differences in their results (*p* > 0.05).
Ingwersen K et al., 2019 [43]	87 patients with chronic shoulder pain. Mean age: G1 = 50.8 ± 12.0; G2 = 50.0 ± 13.4 G1 (n = 43): Intervention group (Psychomotor therapy + active exercise) G2 (n = 44): Control group (active exercise)	Active exercise: 12 weeks. Specific exercises based on the pain and movement restrictions of each patient. Three sets of 15–20 repetitions: strengthening and stabilization exercises for the glenohumeral joint focusing on the rotator cuff muscles and scapula-thoracic muscles. Posture correction and stretching exercises. All exercises were explained by a physiotherapist. Psychomotor therapy: five sessions. Soft manual palpation of muscles, with a focus on shoulder, arm, and neck muscles. Breathing and bodily awareness exercises.	Measurements taken at baseline and at 12 weeks: DASH NRS Pain GPE score	The group that received active exercise + psychomotor therapy had no significant differences in their results compared to the group that only received active exercise therapy (*p* > 0.05).
Gutiérrez–Espinoza H et al., 2019 [44]	80 patients with SIS. Mean age: G1 = 45.2 ± 4.3; G2= 44.5 ± 5.4 G1 (n = 40): Intervention group (specific exercise program + pectoralis minor stretching). G2 (n = 40): Control group (specific exercise program).	Specific exercise program. 12 weeks, 8–10 repetitions for each exercise maintaining the task 5–10 s: conscious control exercises and scapular control exercises. The exercises should be performed painlessly, and mindfully, with progressive loading and focusing on activating weak muscles (SA and LT) and decreasing activation of overactive muscles (UT and deltoids). Pectoralis minor stretching: 10 repetitions of 1 min, in 90° arm abduction and 90° elbow flexion, and with the palmar surface of the hand on the wall. Both groups received six neck and shoulder exercises to perform at home: pain-free active movements of shoulder elevation, shoulder retraction, shoulder abduction in the scapular plane, and neck retraction. Passive stretching of the UT and posterior capsule. Each movement exercise was repeated 10 times and each stretching exercise three times, twice a day at home.	Measurements taken at baseline and at 12 weeks: CMS DASH VAS PMI	Comparison of the results between the two treatments at the end of the 12th week was not statistically significant (*p* > 0.05). DASH questionnaire showed greater functional improvement in the control group. PMI showed a statistically significant difference in favour of the intervention group.
Ribeiro D et al., 2022 [45]	28 patients with shoulder subacromial pain. Mean age: 43.89 ± 9.6; G1 = 43.7 ± 11.7; G2 = 44.1 ± 6.8 G1 (n = 15): standardised exercise group. G2 (n = 13): tailored training group.	16 sessions over 8 weeks with a duration of 60 min. Standardised exercise: eight exercises (progressive resistance training for all scapular and shoulder muscles) + 3 stretches. Tailored training group: Exercises focusing on restoring normal movement patterns and the dynamic stability of the scapulothoracic and glenohumeral joints + manual therapy techniques for restoring shoulder and scapular movement + progressive resistance training of impaired muscles.	Measurements taken at baseline, 4 weeks, 8 weeks, and 12 weeks: Pain: at rest, during movement, and the last week. PSFS SPADI Pain self-efficacy.	There were improvements in both groups but no statistically significant differences were found.
Naranjo-Cinto F et al., 2022 [46]	45 patients with no specific shoulder pain. Mean age: G1 = 30.93 ± 10.87; G2 = 36 ± 15.70; G3 = 35.73 ± 13.66 G1 (n = 15): Sham group (exercise + sham manual therapy on the shoulder and thoracic spine); G2 (n = 15): Thoracic sham (exercise + real manual therapy on the shoulder and sham manual therapy on the thoracic spine); G3 (n = 15): Real MT (exercise + real manual therapy on the shoulder and the thoracic spine);	Two sessions/week for 5 weeks. Therapeutic exercise program: Isometric exercises: shoulder flexion, abduction, internal rotation and external rotation. Three repetitions per exercise with 20 s of contraction, with progressive load and resting 10 s between each repetition. Real manual therapy: - Glenohumeral mobilization technique: three sets of 15 repetitions, 2 Hz of frequency. Rhythmic tractions to the glenohumeral head inducing a flexion-extension movement with the patient in a supine position. - Rib-cage technique: posterior-anterior rhythmic mobilization on the ipsilateral second rib 3 min with a frequency of 2 Hz. Sham manual therapy: the physiotherapist kept their hands in the same place and for the same duration as in the real manual therapy technique but without making any movement.	Measurements taken at baseline, posttreatment, 4-week follow-up, and 12-week follow-up: VAS SPADI	There was a statiscally significant decrease in pain and disability in all groups (*p* < 0.05). There were no statistically significant differences between the different groups.
Alanazi A et al., 2022 [47]	34 patients with SIS Mean age: 39.10 ± 7.94; G1 = 39.15 ± 7.60; G2 = 39.05 ± 8.47 G1 (n = 16): Control (US + stretching exercises + ice). G2 (n = 18): Experimental (handgrip strengthening exercises + US + stretching exercises + ice).	Two sessions/week for 8 weeks: US:3 MHz, 1.5 W/cm^2^ for 8 min. Stretching exercises: posterior shoulder muscle, pectoralis, seated thoracic spine extension, and sleeper stretches. Ten repetitions of 10 s. Handgrip exercises: Three sets of 10 squeezes for 1 min once a day using a heavy-grip hand-gripper. The exercises were performed with the arm at either 30, 60, or 90° of abduction, and with 90° external rotation, adjusting the position of the arm to the patient’s tolerance. Both groups also received a home exercise program once a day for 8 weeks.	Measurements taken at baseline, 4 weeks, and 8 weeks of treatment: VAS DASH SIR SER ROM: FF, A, IR, ER.	Both groups decreased pain and dysfunction but the experimental group (G2) significantly improved shoulder function, pain, strength, and pain-free active range of motion (AROM) compared to control group.

Abbreviations: SIS = subacromial impingement syndrome; GIRD = glenohumeral internal rotation deficit; MCS = modified cross-body stretch; MSS = modified sleeper stretch; VAS = visual analogue scale; CMS = Constant- Murley score; PST = posterior shoulder tightness; IR = internal rotation; ROM = range of motion; ER = external rotation; SPS = shoulder pain syndrome; AROM = active range of motion; SSI = subacromial shoulder impingement; NPRS = numeric pain rating scale; SPADI = shoulder pain and function disability index; SAPS = subacromial pain syndrome; GROC = global rating of change scale; WRNSP = work-related neck-shoulder pain; DASH = disability of arm, shoulder and hand; PBMT = photobiomodulation therapy; SSN = suprascapular nerve; RF = radiofrequency; NHP = Nottingham health profile; C-M = Constant-Murley shoulder assessment score; NRS = Numeric Rating Scale; RCRSP = Rotator cuff-related shoulder pain; PCS = Pain; Catastrophizing Scale; PSEQ = Pain Self-Efficacy Questionnaire; UT = upper trapezius; MT = middle trapezius; LT = lower trapezius; SA = serratus anterior; CPSS = Chronic Pain Self-Efficacy Scale; OSS = Oxford Shoulder Score; NDI = Neck Disability Index; DLA = daily life activities; GPE = Global Perceived Effect; PMI = Pectoralis minor index; PSFS = Patient-Specific Functional Scale; SIR = Strength-Internal rotation; SER = Strength-External rotation; FF = Forward flexion; A = Abduction.

## Data Availability

Not applicable.
